# Lithium: a potential therapeutic strategy in obsessive–compulsive disorder by targeting the canonical WNT/β pathway

**DOI:** 10.1038/s41398-021-01329-3

**Published:** 2021-04-07

**Authors:** Alexandre Vallée, Jean-Noël Vallée, Yves Lecarpentier

**Affiliations:** 1 Department of Clinical Research and Innovation (DRCI), Foch Hospital, 92150, Suresnes, France; 2 Centre Hospitalier Universitaire (CHU) Amiens Picardie, Université Picardie Jules Verne, 80054 Amiens, France; 3 Centre de Recherche Clinique, Grand Hôpital de l’Est Francilien (GHEF), 77100 Meaux, France

**Keywords:** Psychiatric disorders, Biomarkers, Molecular neuroscience

## Abstract

Obsessive–compulsive disorder (OCD) is a neuropsychiatric disorder characterized b–y recurrent and distinctive obsessions and/or compulsions. The etiologies remain unclear. Recent findings have shown that oxidative stress, inflammation, and the glutamatergic pathway play key roles in the causes of OCD. However, first-line therapies include cognitive–behavioral therapy but only 40% of the patients respond to this first-line therapy. Research for a new treatment is mandatory. This review focuses on the potential effects of lithium, as a potential therapeutic strategy, on OCD and some of the presumed mechanisms by which lithium provides its benefit properties. Lithium medication downregulates GSK-3β, the main inhibitor of the WNT/β-catenin pathway. The activation of the WNT/β-catenin could be associated with the control of oxidative stress, inflammation, and glutamatergic pathway. Future prospective clinical trials could focus on lithium and its different and multiple interactions in OCD.

## Introduction

Obsessive–compulsive disorder (OCD) is a neuropsychiatric disorder that affects around 1–2% of the population in their lifetime^1^. OCD is characterized by recurrent and distinctive obsessions and/or compulsions and causes significant problems for patients and their families. OCD is one of the most common mental health disorders in the world^2^. OCD reduces the quality of life, increases the recourse of care services, and impairs social functioning. The presence of mood disorders, depression, anxiety, impulse control disorders, sleep disturbance, and personality disorders could be associated with OCD and exhibit comorbid conditions^3^. These comorbidities can be correlated with social difficulties and can have a major impact on health-related quality of life^4^. The risk of suicide is increased in OCD^5^. Efficacious treatments are needed to face the debilitating nature of OCD^6^. The etiologies of OCD remain unclear, but there are several functional disorders in many structures such as the brain’s orbitofrontal cortex, limbic system, basal ganglia and thalamus, and neurotransmitters^7^.

Nevertheless, the links between neuro-anatomical and biochemical models have not yet been well-established definitively^8^.

In recent years, oxidative stress and free radicals^9^, inflammation^10^, and the glutamatergic pathway^11^ have been shown to play key roles in the causes of OCD.

First-line therapies include cognitive-behavioral therapy^12^. Augmentation strategies with antipsychotics could provide some benefits in at least a third of patients in the case of treatment resistance. Only 40–60% of the patients respond to first-line therapy and research for new treatment beyond current guidelines is mandatory^13^.

This review focuses on the potential effects of lithium, as a potential therapeutic strategy, on OCD and some of the presumed mechanisms by which lithium provides its beneficial properties.

Lithium, which was introduced in 1949, is the main commonly used drug for the treatment of chronic mental illnesses, such as bipolar disorder, characterized by depressive and manic cycles. Lithium remains the first-line therapy for manic-depressive illness, bipolar disorder^14^, traumatic brain injury^15^, and numerous neurodegenerative diseases, such as Alzheimer’s, Huntington’s, and Parkinson’s diseases^16^. In the acute treatment of mania, the efficacy of lithium is well established^17^. Several studies have shown that prophylactically lithium can reduce manic relapses, even if its efficacy is significantly lower in the reduction of depressive relapses^18^. Moreover, other studies have presented that therapy by lithium could reduce suicides and suicide attempts in patients with mood disorders^19^. Lithium therapeutic mechanisms remain complex, including several pathways and gene expression, such as neurotransmitters and receptors, circadian modulation, ion transport, and signal transduction processes^20^.

Thus, recent advances seem to show that the benefits of lithium extend beyond just the treatment of mood. Neuroprotection against excitotoxicity or brain damage is another role of lithium^21^. However, in contrast, several reports have presented that a high dose of lithium could induce irreversible neurotoxicity effects^22^. Excessive intake or impaired excretion could result in lithium accumulation. Lithium is mainly susceptible to accumulation in bone, muscle, liver, thyroid, and kidney^23^. Dehydration, febrile illness, or gastrointestinal loss can lead to elevated lithium levels in serum^24^. Renal toxicity is more common in patients on chronic lithium therapy with nephrogenic diabetes insipidus^25^. The neurologic effects are hyperreflexia, nystagmus, or ataxia and remain mostly reversible^24^. Other troubles are reversible cardiovascular effects (QT prolongation, intraventricular conduction defects)^26^, gastrointestinal effects^27^, and endocrine effects^28^. But, low doses of lithium are correlated with lower side-effects^29^.

### Pathophysiology of OCD

#### OCD and oxidative stress

The oxidative stress process presents an imbalance between production and elimination of reactive metabolites and free radicals (ROS and RNS)^30^. ROS production is due to cell damages by nitration and oxidation of several lipids, proteins, and DNA. The NADPH oxidase (NOX) enzyme involves ROS by the oxidation of intracellular NDAPH to NADP + . Intracellular and extracellular environmental conditions are modulated by ROS production^31^. Mitochondrial dysfunction associated with excessive ROS production and a diminution in ATP production characterize the oxidative stress process^32^. Inflammation markers, such as leukocytes, are recruited from the damage sites and then participate in the increased uptake of oxygen for the release of ROS and thus its accumulation. NOX, activated by the inflammation process, enhances oxidative stress^32,33^.

The main antioxidants are superoxide dismutase (SOD), glutathione peroxides, and catalase. SOD is synthesized in response to oxidative stress and acts as an antioxidant, but its elevation in intracellular conditions increases cell damage by a generation of H_2_O_2_^34^. Glutathione is one of the first-line defense against oxidative stress. Glutathione peroxidases are selenoenzymes that catalyze the reduction in hydroperoxide at the expense of gluthatione^34^. The heme-containing enzyme catalase has a major role in the removal of hydrogen peroxide^35^. They protect bio-membranes against oxidative attack, lipid peroxidation by H_2_O_2,_ and slows down H_2_O_2_-dependent free-radical attack on lipids^36^.

Free radicals (ROS and RNS) induce a decrease in synaptic efficacy^37^ by affecting excitatory and inhibitory synaptic potentials^38^. Free radicals deteriorate membrane lipids by lipid peroxidation, cause ATP depletion, DNA damage and neuronal dysregulation^39^. The brain and nervous system are especially prone to free-radical-induced damage, due to their highly oxygenated organ function^40^ and low catalase activities^41^. The brain presents a large amount of iron and polyunsaturated fatty acids and a moderate amount of SOD and glutathione peroxides^34^. Several studies have shown that free-radical-mediated neuronal dysregulation plays a key role in the pathophysiology of psychiatric diseases by increased SOD activity levels, such as in schizophrenia^42^. The comorbidity observed in OCD raises this possibility of basal ganglia involvement^43^. Major depression presents increased monoamine oxidase activity and elevated antioxidant levels^44^. Recent studies have shown that SOD levels were significantly higher in OCD patients compared to the control group^34^. A higher production of reactive oxygen metabolites, such as the superoxide anion, affects catalase activity^45^, and an increase in production of hydroxyl ions reduces catalase activity^46^. Numerous studies have shown a link between OCD and oxidative stress by the involvement of free radicals and antioxidant defense^34,44^. Moreover, free radicals damage the cell structure and extracellular matrix compounds by disrupting the genetic structure, oxidative stress, mitochondrial dysfunction, and impaired metabolism^9^.

#### OCD and inflammation

Numerous evidence has shown an important role played by the immune system (i.e. inflammation) in the etiology of psychiatric disorders^47^. The link between the immune system and inflammation in OCD pathophysiology is recent and had emerged in the early nineties^11^. Indeed, the pediatric autoimmune neuropsychiatric disorder associated with group A β-hemolytic streptococcus (GABHS) (PANDAS) and thus the recalled pediatric acute neuropsychiatric syndrome (PANS) have highlighted that several agents rather than streptococcus could be involved in these acute-onset forms of OCD^48^. The hypothesis for PANS and PANDAS was a link between gangliosides in basal ganglia neurons and the GABHS and/or other agent^48^. Other studies have presented evidence of inflammatory and immune system increase in pediatric OCD by higher monocytes and CD16 + monocytes compared to healthy control subjects^49^.

Nevertheless, the relevance of neuro-inflammation and autoimmunity in OCD seems not limited to subsets of pediatric and acute onset forms of OCD but could be of interest in adults^50^. The role of inflammation in OCD has been strengthened by the higher rate of anti-basal ganglia antibodies (ABGA) in patients with primary OCD versus control subjects^51^. Moreover, significantly increased levels of cytokines and inflammatory agents have been observed in OCD patients, such as IL-2/4/6/10 and TNF-α, compared to controls^52^. In a recent study using positron emission tomography (PET) imagery, the presence of inflammation in the cortico-striatal-thalamo-cortical circuit was shown to induce microglial cell activation in OCD patients^10^.

#### OCD and microglial dysregulation

Microglia are the brain’s resident immune cells. They are small cells of macrophage lineage originating from hematopoietic progenitors present in the brain. They can be identified in brain tissue by their expression of numerous macrophage markers^53^. Microglia have been presumed to be quiescent under physiological conditions and activated upon immune stimulation. They act in the regulation of neurogenesis^54^, neuronal function, and homeostasis under physiological conditions and in the absence of inflammation^55^. The dysregulated activation of microglia leads to infiltration of the brain by macrophages under pathological conditions^55^. A specific role for microglia in OCD has been suggested in mouse models^56^. However, this mechanism remains unclear.

#### OCD and the glutamatergic pathway

Glutamatergic dysfunction is becoming the principal focus o pharmacological research in the OCD field. Glutamate is an amino acid responsible for the brain’s primary excitatory neurotransmission and is considered as the main neurotransmitter within the cortico-striatal-thalamic circuit involved in OCD^57^. Glutamatergic neurons are embedded in every brain circuit in comparison to dopamine and serotonin, which are used by a small minority of neural cells in the brain. Numerous evidence has shown a glutamatergic dysfunction in OCD^11,58^.

Glutamate is the main excitatory neurotransmitter in the brain and is present in more than 50% of synapses. This signaling plays a major role in neuronal plasticity, memory, and learning^59^. Rapid neurotoxicity enhanced by neuronal excitotoxin has been observed with abnormal glutamate levels^60^.

In neurons, glutamate is stored in synaptic vesicles from which it is released. The release of glutamate leads to increased glutamate concentration in the synaptic cleft to bind ionotropic glutamate receptors. The main consistent candidate gene in OCD is the *SLC1A1* (solute carrier, family 1, member 1) gene^61^. *SLC1A1* encodes for the neuronal excitatory Na + -dependent amino acid transporter 3 (EAAT3). EAAT1 and EAAT2 are the main astrocyte glutamate transporters, whereas EAAT3 is the major neuronal glutamate transporter. Glutamate is converted into glutamine in astrocytes and thus releases it. Then, glutamine is taken up by neurons to be re-converted into glutamate^62^. The role of the EAAT3 is to control glutamate spillover, which affects pre-synaptic *N*-methyl-*D*-aspartate (NMDA) and metabotropic glutamate receptors activity^63,64^. EAAT3 activity is dysregulated by the overexpression of GSK-3β^65^.

Increased levels of glutamate in adult unmedicated patients with OCD have been shown in cerebrospinal fluid (CSF)^66,67^. Moreover, studies based on magnetic resonance spectroscopy (MRS) have observed increased glutamate and related components in brain areas, including central nodes of the cortico-striatal-thalamo-cortical circuit in OCD patients^11,68^. In addition, genetic studies have also involved a correlation of glutamatergic genes with OCD^69^.

### Activation of the canonical WNT pathway by lithium: a potential therapeutic strategy

#### Lithium and GSK-3β

A recent study has observed that mutant murine models of OCD presented increased GSK-3β activity and thus its inhibition could be a treatment of perseverative behaviors^70^.

**Fig. 1:**
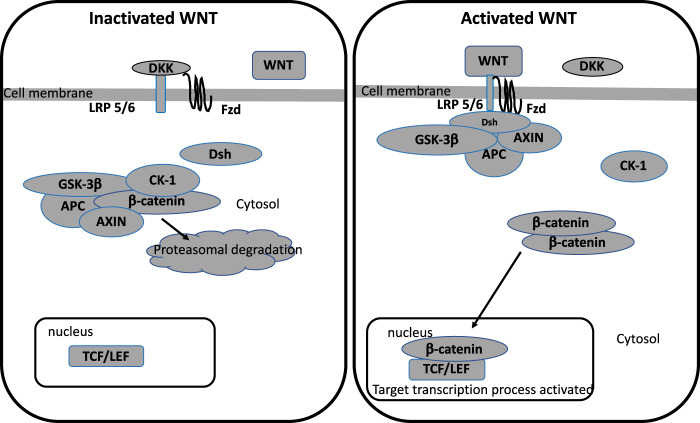
WNT pathway activation and inhibition.

Glycogen synthase kinase-3β (GSK-3β) is a serine/threonine kinase that is involved in numerous intracellular signaling pathways. Dysfunction of GSK-3β is involved in the pathogenesis of several diseases, including neuropsychiatric disorders^71^. GSK-3β is a regulator of several pathways such as inflammation, neuronal polarity, or either cell membrane signaling^72^. GSK3β is known to be the major inhibitor of the canonical WNT/β-catenin pathway^73^. The name WNT is derived from Wingless drosophila melanogaster and its mouse homolog Int. The WNT pathway is involved in numerous signaling and regulating pathways, such as embryogenesis, cell proliferation, migration and polarity, apoptosis, and organogenesis^74^. However, during numerous pathological states, the WNT pathway can be dysregulated, such as in inflammatory, metabolic and neurological disorders, tissue fibrosis, and cancers^75^. GSK-3β downregulates the canonical WNT/β-catenin pathway by inhibiting β-catenin cytosolic stabilization and its translocation in the nucleus^76^. Moreover, several studies have shown a link between neuroinflammation and the augmentation of the GSK-3β activity and in parallel the decrease of the WNT/β-catenin pathway and the protein kinase B (Akt) pathway (Fig. 1)^77^.

Lithium at concentrations of 1–2 mM can inhibit GSK-3β activity^78–80^. Lithium reduces GSK-3β activity by increasing the inhibitory phosphorylation of GSK3β and through direct activation of the Akt pathway. The activation of Akt modulates forkhead bow class O (FOXO)and Bcl-2 associated death protein (Bad) (a pro-apoptotic protein of the Bcl-2 family)^81,82^.

#### Lithium and the WNT/β-catenin pathway

Therapeutic concentrations of the GSK-3β inhibitor lithium lead to the increase in β-catenin levels^83,84^ and then promotes β-catenin transcriptional activity^16,85^. In the brain of a mouse, the over-expression of β-catenin levels mimics the anti-depressant-like effects of lithium^86^, while the knockout of β-catenin leads to a depression-like phenotype^87,88^.

### Lithium in OCD

#### Lithium and oxidative stress

The energy and glucose metabolisms involved during oxidative stress are mainly regulated by the intracellular FOXO transcription factors (FOXO1, 3a, 4)^89^. The interaction between β-catenin and FOXO transcription factors promotes cell quiescence and cell cycle arrest. Β-catenin blocks its transcriptional complex with TCF/LEF through the interaction with FOXO-induced ROS^90^. Β-cateni does not translocate to the nucleus and thus accumulates in the cytosol, leading to the inactivation of the WNT/β-catenin pathway^91,92^. A previous study has found that lithium can reduce FOXO3a transcriptional activity and can decrease the level of active FOXO3a^93^. Thus, by inactivating GSK3-β, activating the WNT/β-catenin pathway, and reducing the FOXO, lithium could participate in the reduction of oxidative stress in OCD.

Furthermore, several in vitro studies have shown that lithium administration could inhibit hydrogen peroxide-induced cell death as well as obstruct lipid peroxidation and protein oxidation in cortical cells^94–99^. Moreover, lithium can act as an anti-oxidant by increasing the CHS levels in neurons of rat dopaminergic N27^95,99^.

#### Lithium and inflammation

**Fig. 2:**
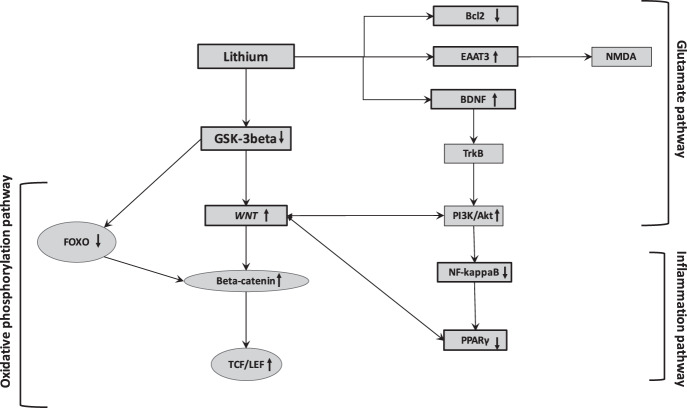
Lithium interactions with oxidative stress, inflammation, and glutamatergic pathways.

Through the inhibition of GSK-3β and thus the upregulation of the WNT/β-catenin pathway, the lithium administration could involve a diminution of the neuro-inflammation by acting on the NF-ϰB pathway. The activation of the WNT pathway cascade restrains inflammation and leads to neuroprotection via interactions between microglia/macrophages and astrocytes (Fig. 2)^100,101^.

Several studies have shown negative crosstalk between the WNT/β-catenin pathway and the NF-ϰB signaling pathway^102^. The NF-ϰB transcription factor family consists of five members in the cytosol under non-activated conditions: NF-ϰB1 (p50/p105), NF-ϰB 2 (p52/p100), RelA (p65), RelB, and c-Rel^103^. Β-CATENIN can form a complex with RelA and p50 to decrease the activity of the NF-ϰB signaling^104^. Moreover, by interacting with the PI3K, β-catenin inhibits the functional activity of NF-ϰB^105^. This inhibitory function of β-catenin on NF-ϰB activity has been observed in numerous cell types, such as fibroblasts, epithelial cells, hepatocytes, and osteoblasts^102^. In parallel, the overactivation of GSK-3β leads to an inhibition of the β-catenin and then activation of the NF-ϰB pathway^106^. The potential protective action of β-catenin was due to the activation of the PI3K/Akt pathway and thus the reduction of TLR4-driven inflammatory response in hepatocytes^107^. NF-ϰB activation leads to the inhibition of the complex β*-*catenin/TCF/LEF by the upregulation of LZTS2 in cancer cells^108^. DKK, a WNT inhibitor, was a target gene of the NF-ϰB pathway leading to a negative feedback to diminish the β-catenin signaling^109^.

A recent study has presented that the WNT pathway appeared to be one of the main mechanisms of the action of lithium in adipose cells, and this interaction is done by the inhibition of PPARγ expression^110^. PPARs are ligand-activated transcription factors that bind PPRE (PPAR-response elements). PPARs are involved in numerous pathophysiological processes, such as cell differentiation, protein metabolism, lipids metabolism, carcinogenesis^111,112^, adipocyte differentiation, insulin sensitivity, and inflammation^113,114^. PPARγ ligands, such as thiazolidinediones (TZDs), are able to decrease inflammatory activity^115^.

A negative crosstalk has been well described between PPARγ and the WNT pathway^32,73,116,117^. The PI3K/Akt pathway, which is positively induced by β-catenin^118,119^, acts by phosphorylating GSK-3β to negatively regulate PPARγ expression^120^. PPARγ agonists decrease β-catenin expression by overactivating GSK-3β^121^. Moreover, PPARγ agonists activate Dickkopf-1 (DKK1) activity to decrease the canonical WNT/β-catenin pathway and then inhibit fibroblast differentiation^122^. Furthermore, PPARγ agonists activate GSK-3β to decrease β-catenin expression^121^.

#### Lithium and the glutamatergic pathway

Lithium administration has been also associated with an influence on the levels of proapoptotic proteins. Bax, named Bcl-2 associated C protein, is a key modulator promoting apoptosis by binding to and antagonizing the Bcl-2 protein. The tumor suppressor protein, p53, targets Bcl-2 and Bax and then promotes growth arrests and cell death in response to cell damage (Fig. 2)^123^.

Several studies have demonstrated that the neuroprotective effects of lithium could be attributed to increased Bcl-2 levels. Indeed, lithium therapy of cultured cerebellar granule cells increased mRNA and protein levels of Bcl-2, and the Bcl-2/Bax protein level ratio increased by 5-fold after treatment duration for 5–7 days^124^. The increase in Bcl-2 expression leads to neurogenesis in the hippocampus and entorhinal cortex in mice by the increase of axon diameters and neurite growth on the CA3 area of the hippocampus and increased myelination in the entorhinal cortex^125^. Lithium can also act by stimulating anti-apoptotic-increasing Bcl-2 levels and reducing Bax^126^. The phosphorylation of Bcl 2 at serine 70 is needed for a complete anti-apoptotic function^127^ and lithium has this ability^128^. Lithium inhibits Bcl-2 dephosphorylation and caspase-2 activation through the reduction of the protein phosphatase-2A activity^128^.

Glutamate excitotoxicity has been associated with the upregulation of Bax and p53 and the downregulation of Bcl-2^124^. The apoptosis attributed to glutamate was preceded by the increase in activator protein-1 (AP-1) caused by the activation of c-Jun N-terminal kinase (JNK) and p38 mitogen-activated protein kinase (MAP kinase) and phosphorylation of c-Jun and p53^129^.

By inhibiting GSK-3β activity, lithium acts as a powerful regulator of EAAT3 and thus of the regulation of NMDA receptors^130^. Moreover, a direct potential way could be the inhibition of presynaptic NMDA receptors and thus the activation of postsynaptic AMPA receptors by the release of glutamate. This mechanism is followed by the activation of the influx of calcium and secretion of brain-derived neurotrophic factor (BDNF). Lithium stimulated the release of the excitatory neurotransmitter, glutamate, from cerebral cortex slices^131^. This release was accompanied by an increase in inositol 1,4,5-trisphosphate [Ins(1,4,5)*P*_3_] accumulation. The increase in Ins(1,4,5)*P*_3_ accumulation was caused by the selective activation of the N-methyl-D-aspartate (NMDA) receptor/channel by glutamate. Activation of the NMDA receptor is known to cause increased Ins(1,4,5)*P*_3_ accumulation^132^. Thus, BDNF stimulates the receptor tyrosine kinase B (TrkB), leading to neuronal survival and differentiation^133^.

Activated BDNF-TrkB signaling leads to stimulation of the Akt/mTOR pathway, causing activation of the WNT/β-catenin pathway and enhancing synaptic proteins^134^. The few therapeutic levels of lithium activate the BDNF-TrkB signaling and then the Akt/mTOR signaling to protect neurons from glutamate excitotoxicity^135^. Lithium inhibits excessive glutamate, NMDA receptor-mediated calcium influx in neurons and reduces NR2B subunit tyrosine phosphorylation by the Src/Fyn kinase^136^.

PPARγ antagonists can block the increase of PPARγ DNA binding activity and antioxidant enzymatic activities (SOD), inhibiting the protection of PPARγ activation in OGD-exposed neurons^137^. Other mechanisms by which these PPARγ agonists prevent oxidative stress include a decrease in iNOS activity, NFκB blockade, inhibition of TNF-α release, or activation of nuclear factor (erythroid-derived 2)-like 2 (Nrf2)^138^. By the negative crosstalk between WNT and PPARγ, lithium administration, by inhibiting the GSK-3β could act as a PPARγ antagonist and lead to an increase in the WNT pathway, resulting in diminution of oxidative stress.

## Conclusion

Currently, few studies have studied lithium as a possible alternative therapeutic way to treat OCD patients. However, in low doses, lithium may appear to be interesting against OCD because of its potential inhibitory effect on oxidative stress, inflammation, and the glutamatergic pathway.

No study has still reported the expression of the WNT/β pathway in OCD. Nevertheless, the overactivity of the GSK-3β, the main inhibitor of the WNT pathway, in OCD patients is consistent with a downregulation of the WNT pathway in this disease. By stimulating the WNT/β pathway, through the inhibition of GSK-3β, lithium could be an innovative therapeutic way in OCD. Future prospective studies could focus on lithium and its different and multiple interactions in OCD.
